# Densely Stacked CoCu-MOFs Coated with CuAl/LDH Enhance Sulfamethoxazole Degradation in PMS-Activated Systems

**DOI:** 10.3390/nano15060432

**Published:** 2025-03-11

**Authors:** Xin Zhong, Xiaojun Liu, Meihuan Ji, Fubin Jiang

**Affiliations:** 1 Experimental Education Platform, Beijing Normal University at Zhuhai, Zhuhai 519087, China; meihuanji@bnu.edu.cn; 2 Faculty of Arts and Sciences, Beijing Normal University at Zhuhai, Zhuhai 519087, China; xiaojunliu@bnu.edu.cn (X.L.); jfb@bnu.edu.cn (F.J.)

**Keywords:** PMS activation, antibiotics, CuAl/LDH, CoCu-MOFs

## Abstract

As the most promising techniques for refractory antibiotic degradation in wastewater management, sulfate radical-based advanced oxidation processes (SR-AOPs) have attracted considerable attention. However, systematic studies on potassium peroxymonosulfate (PMS) activation by MOF-derived metal oxides coated with LDH materials are still lacking. In this work, a series of catalysts consisting of CoCu-MOFs coated with CuAl/LDH were synthesized for PMS activation in the removal of sulfamethoxazole (SMX). As expected, CoCu-MOFs coated with CuAl/LDH catalyst showed high SMX removal and stability in PMS activation. In the CoCu/LDH/PMS reaction, the SMX removal was nearly 100% after 60 min, and the mineralization reached 53.7%. The catalyst showed excellent catalytic stability and low metal leaching concentrations (Co: 0.013 mg/L, Cu: 0.313 mg/L), as detected by ICP. Sulfate radicals and hydroxyl radicals were identified as the dominant reactive species in the CoCu/LDH/PMS system. Moreover, the presence of ^1^O_2_ in the process revealed the coupling of non-radical and radical processes. The XPS results showed that the layered structure of CoCu/LDH promoted the recycling of metal ions (high and low valence), which facilitated heterogeneous PMS activation. The effects of different reaction conditions and reuse cycles were also determined. The SMX oxidation pathways were proposed based on the intermediates identified by LC/MS. The high activity and stability of CoCu/LDH provide a new mechanistic understanding of PMS activation catalysts and their potential utilization in practical wastewater treatment.

## 1. Introduction

With the rapid development of the pharmaceutical industry, residual antibiotic compounds are recognized as emerging pollutants [1]. The threat to human health and society already exists [2,3]. Residual antibiotic compounds usually possess poor biodegradability and complex structures, which make them difficult to remove from the aquatic environment [4]. As one of the anti-biodegradable antibiotics, sulfamethoxazole (SMX) has been widely used in animal husbandry, leading to emerging residues in ground water [5,6,7]. It can also suppress Staphylococcus and Escherichia coli, which have been detected in municipal sewage and wastewater [8,9,10]. Excessive accumulation of SMX can cause serious environmental problems, meaning that it is necessary to reduce the residual concentration to an acceptable level [11,12,13,14]. Concerning the safety of drinking water, conventional water treatment technology presents limitations for the removal of SMX [15]. Therefore, efforts to find other available technology to remediate these refractory chemicals in wastewater have been made.

Among the various advanced oxidation processes (AOPs), sulfate radical-based AOPs (SR-AOPs), with the formation of sulfate radicals (SO_4_^•−^), have received attention due to their high catalyst activity, high oxidation potential, and long half-life in the removal of refractory chemicals [16]. As the two main sources of sulfate radicals, potassium peroxymonosulfate (PMS, HSO_5_^−^) and peroxydisulfate (PS, S_2_O_8_^2−^) can be effectively stimulated by metal ions, alkaline, carbon materials, and transition homogeneous and heterogeneous metals catalysts [17,18,19,20]. PMS was considered as an excellent precursor to generate sulfate radicals owing to its asymmetric structure [21]. Sulfate radicals exhibited excellent performance due to their high oxidation potential (2.5–3.1 V), long half-life (30–40 μs), and validity in a wide pH range, resulting in good catalytic selectivity and stability in wastewater treatment [22]. Extra energy, such as UV, heat, and ultrasound, are also employed to activate PMS to generate sulfate radicals [23]. However, the complicated equipment, time-consuming operation, and energy requirements limit the practical application in water treatment. Alternatively, PMS activation based on transition metal-based heterogeneous catalysts effectively avoids the aforementioned drawbacks [24]. Among them, cobalt-based catalysts were the most valuable catalysts, while the application of cobalt-based catalysts was limited by the leaching problems of toxic cobalt ions [25,26,27]. Based on this, more studies have focused on transition metal-based bimetallic catalysts, taking great advantage of their higher catalytic activity and lesser leaching problems [28]. Copper is considered to be a good partner for Co, which cannot only promote the redox cycle but also increases the degradation rate with limited cobalt ions leaching problems [29].

On the other hand, metal-organic framework (MOF) materials were considered as an good PMS activator due to their high porosity, high specific surface area, and controllable structure [30,31,32]. By modifying the MOF skeleton, it is feasible to design and control the crystallinity, void space, and surface activity of the material, which showed excellent properties for PMS activation due to its unique structure [33,34]. Moreover, layered double-metal hydroxides (LDHs) consisting of interlayer anions and metal hydroxides have also proven a popular precursor for PMS activation [35]. The acid and base sites on the LDH layer can control pH drift in the solution, which maintains the pH during the reaction and mitigates sudden changes in pH [36]. The LDHs consisted of positively charged metal laminates and interlayer anions [37]. For example, Cu_2_Fe_0.5_Al_0.5_-LDH catalyst was synthesized to activate PMS for the degradation of SMX and showed excellent catalytic activity [38]. The hybridization of MOFs with LDHs as precursors to fabricate composites was successful for promoting the cleavage of PMS bonds and the formation of free radicals, resulting in increased catalytic activity. By introducing Co-Cu oxides into the outer layer of the LDH shell, a core-shell-like structure can be established [29]. The catalyst stability was improved, while the hetero-structured materials inherited the initial features of the precursors. The active sites were dispersed throughout the layered structure owing to the introduction of the outer shell. The high-porosity structure facilitates the reaction of reactive species with pollutants, leading to high catalytic activity. To extend the full application range of MOF-based and LDH-based materials, various valuable materials were used as the heterogeneous catalyst for PMS activation, as listed in Table S1.

Inspired by this, the CoCu-MOFs coated with CuAl/LDH catalyst were prepared via a facile strategy for the PMS activation process. The physical-chemical properties and morphology of CoCu-MOFs and CoCu/LDH were studied. In addition, the effects of various reaction conditions on SMX removal were studied to determine the universality of the catalyst. The mechanism of PMS activation was evaluated by radical quenching experiments and electron paramagnetic resonance (EPR). The intermediates produced during SMX degradation were detected via liquid chromatography-mass spectrometry (LC/MS), and the degradation pathways were elucidated. The reusability of the catalyst was evaluated to determine actual application potential of CoCu-MOFs and CoCu/LDH. This research offers creative ideas regarding the high catalytic activity of CoCu-MOFs and CoCu/LDH in the decomposition of PMS, further elucidating the mechanism of CoCu/LDH/PMS reactions in actual wastewater.

## 2. Materials and Methods

### 2.1. Chemicals

Cobalt nitrate (Co(NO_3_)_2_·6H_2_O), copper nitrate (Cu(NO_3_)_2_⋅3H_2_O), and aluminum nitrate (Al(NO_3_)_3_⋅9H_2_O) were obtained from Xilong Chemical Co., Ltd. (Guangzhou, Guangdong, China). 2-aminoterephthalic acid (NH_2_-BDC) was supplied by Shanghai Macklin Biochemical Technology Co., Ltd. (Shanghai, China). Sulfamethoxazole (SMX) and potassium peroxymonosulfate (PMS) were obtained from Xilong Scientific Technology Co., Ltd. (Shenzhen, China). Methanol (MeOH), L-histidine, tert-butanol (TBA), Orange II, and phenol were purchased from Shanghai Aladdin Biochemical Technology Co., Ltd. (Shanghai, China). All chemicals were of analytical grade and were used without further purification. All aqueous solutions were prepared with ultrapure water produced with a water purifier in the laboratory.

### 2.2. Preparation of CoCu-MOFs and CoCu/LDH

Synthesis of CoCu-MOFs. The CoCu-MOFs were synthesized via a hydrothermal method by mixing Co(NO_3_)_2_⋅6H_2_O (0.75 mmol) and Cu(NO_3_)_2_⋅3H_2_O (1.5 mmol) with 10 mL of ethanol. Then, NH_2_-BDC (1.5 mmol) and 40 mL of N, N-dimethylformamide (DMF) were added to the mixture under vigorous stirring. The mixture was carefully transferred to a Teflon-lined stainless-steel autoclave, which was sealed and heated at 120 °C for 10 h. Then, the nanoparticles were centrifuged and washed with DMF several times. The particles were subsequently dried at 60 °C for 8 h to obtain CoCu-MOFs.

Synthesis of CoCu/LDH. First, certain amounts of Cu(NO_3_)_2_⋅3H_2_O (2 mmol) and Al(NO_3_)_3_⋅9H_2_O (1 mmol) were dissolved in 20 mL of deionized water to obtain solution I. Then, NaOH and Na_2_CO_3_ were dissolved in 15 mL of deionized water to obtain solution II. Solution I and solution II were mixed with CoCu-MOFs nanoparticles under magnetic stirring for 8 h. Then, the mixture was transferred to a Teflon-lined stainless autoclave, which was sealed and heated at 120 °C for 10 h. Finally, the samples were centrifuged and washed with deionized water. The resultant material was dried at 60 °C for 8 h to obtain CoCu/LDH.

### 2.3. Characterization

Characterization of the catalysts was carried out by X-ray powder diffraction (XRD, Smartlab SE, Tokyo, Japan), X-ray photoelectron spectroscopy (XPS, Nexsa G2. Thermo Fisher, Waltham, MA, USA), and Fourier transform infrared spectroscopy (FTIR, Nicolet iS5, Thermo Fisher, Waltham, MA, USA). The morphology of the samples was investigated by scanning electron microscopy (SEM, Thermo Fisher Scientific Apreo 2S, Waltham, MA, USA) and transmission electron microscopy (TEM, JEM F200, Tokyo, Japan). EPR was performed to detect reactive oxygen species on a Bruker X-band spectrometer (Billerica, MA, USA) with DMPO and TEMP as the spin-trapping agents.

### 2.4. Degradation Experimental Procedure and Analytical Methods

SMX was selected as the target pollutant to evaluate the catalytic performance of CoCu/LDH in PMS activation. First, the catalyst was added to 100 mL of SMX solution (15 mg/L). Then, a certain amount of PMS was added to the system. At various time intervals, a 1.0 mL sample was taken and filtered through a 0.22 µm membrane to analyze the concentration of SMX.

The SMX concentration was measured by a high-performance liquid chromatography (HPLC) instrument (LC 20, 0.1% acetic acid/acetonitrile = 0.4/0.6, C18 column). Other contaminants, such as Rhodamine B (RhB), Orange II, and phenol, were detected by UV-vis spectrometer (Nanjing Feile Instrument Co., Ltd., Nanjing, China) to verify the universality of the catalyst. To evaluate the reusability of the catalyst, the catalyst was filtered, washed with ultrapure water and then dried in an oven at 60 °C. The concentration of metallic ions in the aqueous medium was determined by inductively coupled plasma (ICP) (Thermo Fisher Scientific, Waltham, MA, USA). The degradation intermediates were identified by LC/MS (Waters Q-TOF, Milford, CT, USA), and a degradation pathway was proposed. Radical quenching experiments were carried out with MeOH, TBA and L-histidine to trap sulfate radicals, hydroxyl radicals, and singlet oxygen. The total organic carbon (TOC) content was measured by a TOC analyzer (HM-TOC1, Weifang, China). The SMX degradation rate constants were calculated and fitted with a first-order kinetic model.

## 3. Results and Discussion

### 3.1. Catalyst Characterization

**Figure 1:**
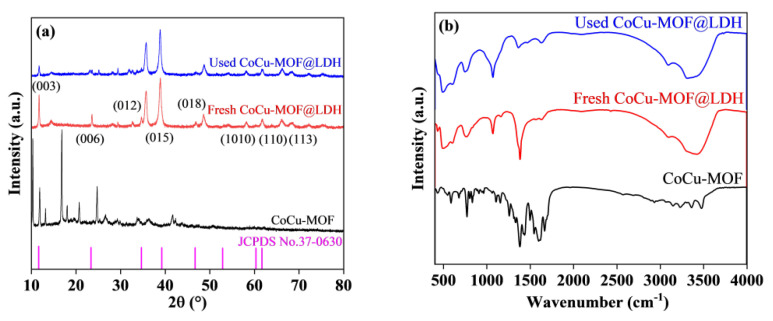
(**a**) XRD spectra of synthesized CoCu‒MOFs and fresh and used CoCu/LDH. (**b**) FTIR spectra of synthesized CoCu-MOFs and fresh and used CoCu/LDH.

The XRD patterns of CoCu-MOFs and CoCu/LDH are depicted in Figure 1. All the diffraction peaks were sharp, demonstrating the good crystallinity of the synthesized materials. The peaks at 18.8°, 21.2°, 28.1°, 29.6°, 34.1°, 35.9°, 36.7°, 38.8°, and 40.4° corresponded to (002), (101), (010), (011), (012), (110), (103), (004), and (013) of Cu_2_CoO_3_ (JCPDS No. 21-0288). Meanwhile, extra peaks at 18.7°, 30.9°, 36.4°, 38.1°, and 42.3° could be observed in the samples of Co_3_O_4_ and CoO reflections. The other peaks at 24.5°, 25.8°, 33.1°, 35.2°, and 42.2° were ascribed to the phases of CuO reflections. Thereby, the CoCu-MOFs could have multiple oxide compositions, including the Co and Cu oxides. Meanwhile, the peaks at 11.61°, 23.35°, 34.66°, 39.2°, 46.69°, 52.85°, 60.32°, and 61.66° corresponded to the (003), (006), (012), (015), (018), (1010), (110), and (113) planes of CuAl/LDH, respectively (JCPDS No. 37-0630). The peak intensities for CoCu/LDH decreased slightly due to coating with the LDH shell, which suggested that much less metal leaching would occur during further PMS activation. However, the catalyst also displayed the peak of copper oxide, which led to excessive copper leaching during the catalytic process. The copper leaching is 0.313 mg/L and the cobalt leaching is 0.013 mg/L, as detected by ICP measurement. Reused CoCu/LDH maintained the same main structure as the fresh catalyst, indicating good reusability in the following reaction sequence.

FTIR was performed to identify the chemical structures of CoCu-MOFs and CoCu/LDH, and the resulting spectra are shown in Figure 1b. There was a broad adsorption band for the CoCu-MOFs and CoCu/LDH catalysts, ranging from 3180 to 3560 cm^−1^, which was attributed to the stretching vibration of hydroxyl groups and N–H bond. The vibration peaks located at 678.2 and 579.3 cm^−1^ was attributed to Co–O and Cu–O bonds. The peaks at 1385.5 and 1636.9 cm^−1^ were attributed to the deformation vibration of –OH and carbonate, which were attributed to water molecules and carbonate ions in the LDH layer. Moreover, the stretching vibration peaks of Al–OH, Al–O, and Cu–OH were observed at 773.4, 606.8, and 837.2 cm^−1^, respectively. Notably, the intensities of the Co–O and Cu–O peaks were much weaker in the FTIR spectrum of CoCu/LDH than those of the other materials due to the core-shell structure. The peak intensity corresponding to –OH stretching vibrations in the used catalyst was much greater than that in the fresh catalyst, which was caused by adsorption during the catalytic process.

**Figure 2:**
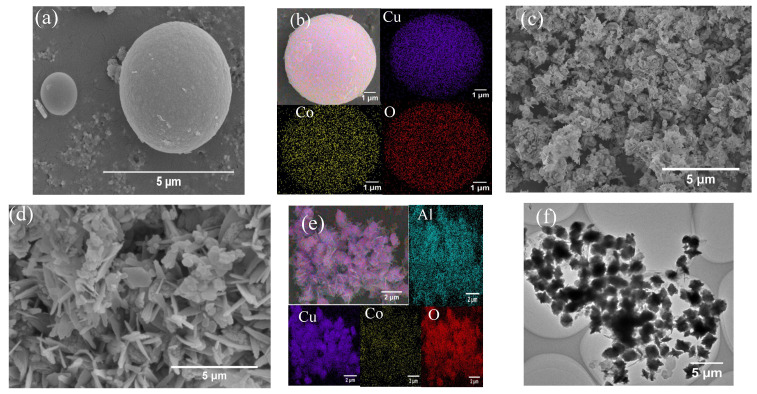

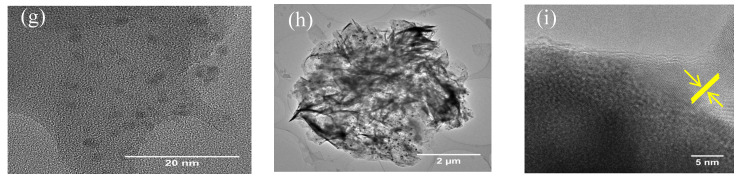
(**a**,**b**) SEM images and EDS mapping images of CoCu-MOFs. (**c**–**e**) SEM images and EDS mapping images of CoCu/LDH. (**f**) TEM images of CoCu-MOFs. (**g**–**i**) TEM images of CoCu/LDH.

The morphologies of CoCu-MOFs and CoCu/LDH are displayed in Figure 2a–e. CoCu-MOFs exhibited a smooth and spherical morphology, with a sphere diameter approaching 5.7 μm. CoCu/LDH (Figure 2e) had a sheet structure with confined spherical CoCu-MOFs in the layers. The SEM-EDS mapping results (Figure 2b,e) revealed the average distributions of Co, Cu, Al, and O in the material, which were close to the theoretical ratios. TEM images of CoCu-MOFs and CoCu/LDH were taken. As displayed in Figure 2g, the fine CoCu-MOFs particles was successfully wrapped within the layered LDH. Figure 2h shows the uniform distribution of elements on the surface. The CoCu/LDH layers were nearly nanoscale in size, oriented in different directions, and densely stacked, which could facilitate repeated catalytic performance. The high-resolution transmission electron microscopy (HRTEM) images revealed lattice fringes of 0.62 nm, corresponding to the lamellar structure of the LDH.

### 3.2. Catalytic Degradation Activity and Properties

**Figure 3:**
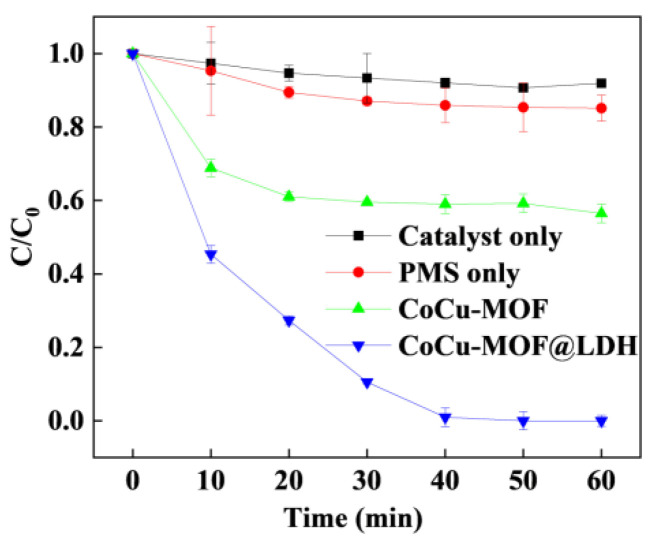
The degradation of SMX in different degradation processes. Experiment condition: [SMX] = 15 mg/L, [catalyst] = 0.1 g/L, and [PMS] = 0.4 mM.

The catalytic degradation performance of different systems in PMS activation for SMX degradation was investigated, and the results are shown in Figure 3. The SMX adsorption was only 9.2% after 60 min; thus, the effects of SMX adsorption could be neglected in the reaction sequence. The SMX removal was almost none in the PMS-only reaction, as there were almost no reactive oxidant species. In the catalyst + PMS system, the SMX removal rates of CoCu-MOFs and CoCu/LDH were 58.8% and 98.3%, respectively, and the corresponding reaction rate constants were 0.1567 min^-1^ and 0.2026 min^−1^. The results revealed more valuable PMS activation in the presence of CoCu/LDH. Moreover, the concentration of Co ions in solution was determined by ICP, which was only 0.013 mg/L, indicating excellent catalytic stability. In addition, Al was not detected in the solution, and the concentration of Cu ions was 0.313 mg/L, which is lower than the drinking water standard limit for China. The TOC removal was approximately 53.7% after a 60 min reaction. The results showed that heterogeneous catalysis was the main PMS activation process rather than the activation of homogeneous metal ions. In conclusion, confined Co-Cu particles in the layered LDH shell offered more active sites for PMS activation, indicating that the core-shell structure facilitated PMS activation for SMX removal and reduced the metal leaching during the reaction.

### 3.3. Effects of Different Parameters on SMX Degradation

**Figure 4:**
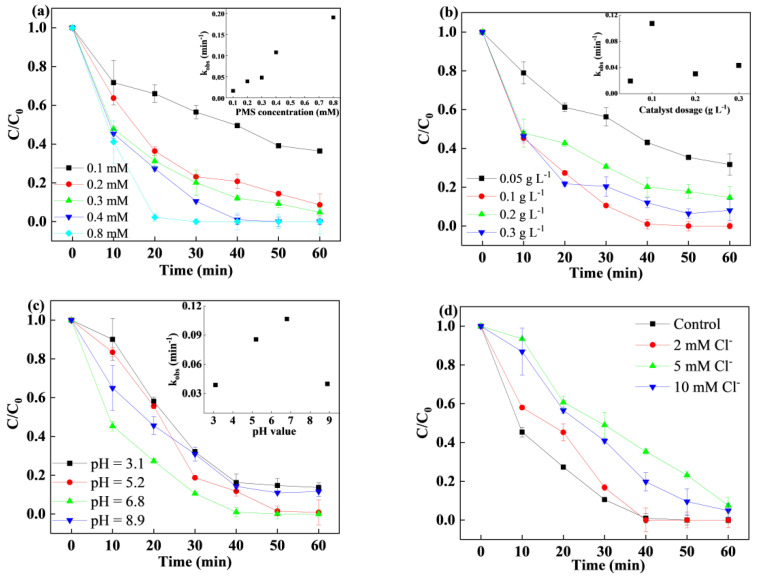

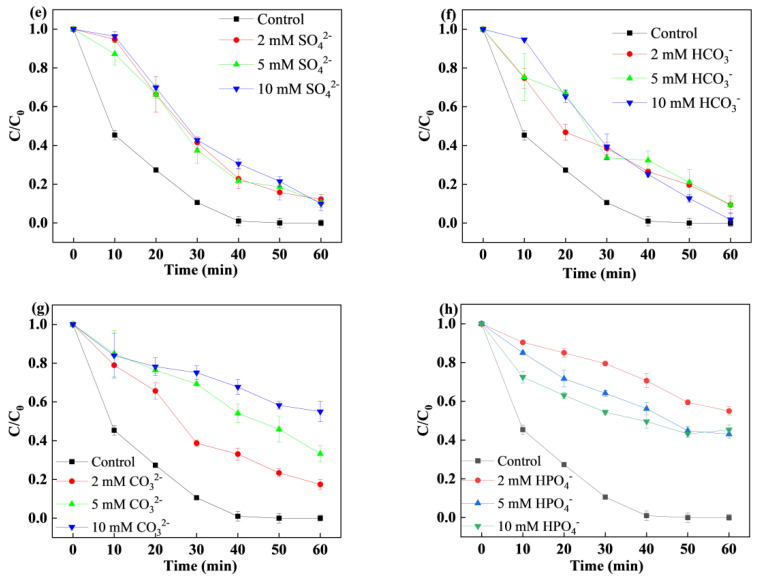
(**a**) Effect of PMS concentration. (**b**) Effect of catalyst dosage. (**c**) Effect of initial pH value. (**d**) Effect of Cl^−^ concentration. (**e**) Effect of SO_4_^2−^ concentration. (**f**) Effect of HCO_3_^−^ concentration. (**g**) Effect of CO_3_^2−^ concentration. (**h**) Effect of HPO_4_^−^ concentration. Experiment conditions: [SMX] = 15 mg/L, [catalyst] = 0.1 g/L, and [PMS] = 0.4 mM.

Effect of PMS and catalyst dosage. It is known that reactive free radicals are closely related to the degradation rate of target pollutants [39]. In this case, the effect of PMS concentrations in the range of 0.1 mM to 0.8 mM was evaluated, as shown in Figure 4a. The SMX removal and rate constant both increased with increasing PMS concentration but showed no further enhancement at excessive PMS dosages. With increased PMS dosage, the SMX removal increased from 63.6% to 100%, indicating that more free radicals were generated as the PMS dosage increased. In addition, the kinetic removal constants did not differ between 0.04 mM and 0.08 mM, which could be attributed to the competitive reaction between free radicals and PMS. Based on economic and environmental considerations, 0.4 mM PMS was chosen for subsequent experiments. Additionally, the SMX removal increased significantly from 68.3% to 100% as the catalyst dosage increased from 0.05 g/L to 0.1 g/L, which was attributed to the increased number of available active sites. However, as the catalyst dosage further increased to 0.1 g/L, the SMX removal declined to 85.3%. Decreased SMX removal was also found in previous studies on catalyst/PMS systems. The reasons as follows: (i) the formed sulfate radicals could induce a self-quenching reaction with increasing catalyst dosage, leading to the inhibited removal; (ii) excess catalyst could react with generated radicals due to the scavenging effect; and (iii) a high degree of turbidity due to excessive catalyst in suspension would limit contact with PMS [40,41,42]. When the PMS content remained constant, a certain number of catalyst active sites was sufficient for PMS activation. The kinetic constant of SMX degradation first increased from 0.0193 min^−1^ to 0.1067 min^−1^ when the catalyst dosage increased from 0.05 g/L to 0.1 g/L and then slightly decreased to 0.0432 min^−1^ when the catalyst dosage further increased to 0.3 g/L. For the above reasons, the optimal catalyst dosage was 0.1 g/L.

^•^OH + ^•^OH → H_2_O_2_ (1)

SO_4_^•ࢤ^ + ^•^OH→ HSO_5_^−^ (2)

Effect of initial pH and inorganic anions. The effect of initial pH was investigated in the range of 3.1 to 8.9, as shown in Figure 4c. The SMX removal was inhibited in acidic solutions, while when the pH was raised to 6.8, the SMX removal increased from 86.7% to 100%. Then, it slightly dropped to 88.4% as the pH further increased to 8.9. Moreover, the rate constant first increased from 0.0388 min^−1^ to 0.1067 min^−1^ as the pH value was raised from acidic to neutral and then decreased to 0.04 min^−1^ when the pH was close to 8.9. The SMX removal remained high within a wide pH range (3–9) but was slightly inhibited under alkaline and acidic conditions. When the solution pH increased from 3.1 to 6.8, the catalyst was positively charged, resulting in electrostatic attraction and greater contact with SMX. Moreover, it was proven that PMS was present mainly in the form of HSO_5_^−^ under acidic conditions, leading to decreased SMX removal [43]. On the other hand, the outer LDH shell could provide a unique buffer capacity in the solution, resulting in similar removal levels over a wide pH range. When the solution was under alkaline condition, PMS would undergo self-decomposition, resulting in the production of less reactive species to react with SMX [44]. In addition, excess OH^−^ could react with Cu and Co species to generate hydroxide precipitates on the catalyst surface, which leads to fewer reactive sites participating in the catalytic reaction. Under acidic and neutral conditions, Cu and Co species played a dominant role and could effectively react with PMS, leading to the formation of free radicals. H^+^ could also accelerate the metal leaching problem, which was much higher under acidic conditions.

It is important to investigate the effects of inorganic anions on SMX degradation for subsequent practical application [45]. The influence of inorganic anions, such as Cl^−^, SO_4_^2−^, HCO_3_^−^, CO_3_^2−^ and HPO_4_^−^, at concentrations of 2, 5, and 10 mM, was studied in the CoCu/LDH/PMS reaction. The results showed that Cl^−^ hindered SMX removal, and the SMX removal decreased to 92.51% with the addition of 5 mM NaCl. Cl^−^ could participate in the generation of sulfate radicals, which promoted removal. Moreover, self-quenching reactions occurred in the system, resulting in decreased SMX removal [46]. The SMX removal with the addition of 10 mM NaCl was greater than that with the addition of 5 mM NaCl, which was attributed to the interaction of Cl^−^ and PMS leading to the formation of ^•^Cl and ^•^HOCl. In the presence of SO_4_^2−^, the SMX removal was slightly decreased, which was attributed to low reactivity with free radicals. The interaction between SO_4_^2−^ and SO_4_^·−^ reduces the amount of reactive species, leading to decreased catalytic activation in heterogeneous systems. HCO_3_^−^, CO_3_^2−^, and HPO_4_^−^ clearly inhibited SMX removal, and the inhibitory effect became greater as the concentration of inorganic ions increased. The results showed that both HCO_3_^−^ and CO_3_^2−^ were effective scavengers of sulfate radicals in the system, especially in the presence of CO_3_^2−^. Owing to the unique core-shell structure of LDH, the pH of the solution was maintained during the catalytic reaction, and the addition of HCO_3_^−^ and CO_3_^2−^ barely changed the pH of the solution. The presence of HCO_3_^−^ and CO_3_^2−^ resulted in side reactions, which reduced the amount of active species in solution and negatively impacted the SMX removal. The SMX removal decreased to 40.3% in the presence of 10 mM HPO_4_^−^. Less reactive phosphate radicals were produced by HPO_4_^−^, sulfate radicals, and hydroxyl radicals. In addition, HPO_4_^−^, copper ions, and cobalt ions could react and generate chemical complexes on the catalyst’s surface, leading to decreased catalyst activity.

Cl^−^ + HSO_5_^−^ → SO_4_^2−^ + HClO (3)

Cl^−^ + SO_4_^•−^→ ^•^Cl + SO_4_^2−^ (4)

Cl^−^ + ^•^OH → ^•^ClOH (5)

^•^ClOH + H^+^ → ^•^Cl + H_2_O (6)

^•^OH + HCO_3_^−^ → CO_3_^•−^ + H_2_O (7)

SO_4_^•−^ + HCO_3_^−^ → CO_3_^•−^ + SO_4_^2−^ + H^+^ (8)

### 3.4. Stability and Reusability of CoCu/LDH

**Figure 5:**
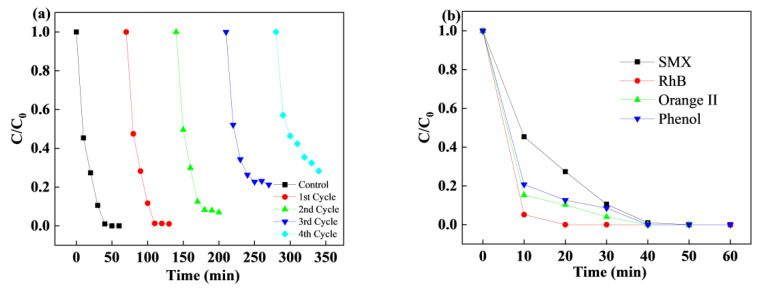
(**a**) Reusability of CoCu/LDH for SMX degradation. (**b**) The SMX removal of various target pollutants in CoCu/LDH/PMS system. Experiment conditions: Experiment conditions: [SMX] = 15 mg/L, [Orange II] = [phenol] = [RhB] = 15 mg/L, [catalyst] = 0.1 g/L, and [PMS] = 0.4 mM.

The stability and reusability of a catalyst are important factors for evaluating actual application [47]. The SMX removal remained high with the same PMS and catalyst dosages, but the SMX removal declined from 100% to 91.4% in the recycle test, as shown in Figure 5a. The decrease was attributed to the adsorbed intermediates on the surface of the catalyst, resulting in less contact with the target pollutants. The maintained SMX removal degree demonstrated good catalytic performance in heterogeneous processes. The good crystallinity and high intensity of the characteristic peaks of the reused catalyst were unchanged, demonstrating the stability of the catalyst. Moreover, as detected by ICP, the concentrations of leached copper and cobalt ions were 0.313 and 0.013 mg/L, respectively. Furthermore, to investigate the universality of the catalyst, different target pollutants, including Orange II, RhB, and phenol, were chosen for the CoCu/LDH/PMS process. Orange II and RhB were removed completely within 20 min, while the phenol degradation reached 100% in 60 min which was shown in Figure 5b. The recycling performance of CoCu/LDH further confirmed that the simple preparation method can be used to generate catalysts with high reusability and stable catalytic activity.

### 3.5. Degradation Mechanism

**Figure 6:**
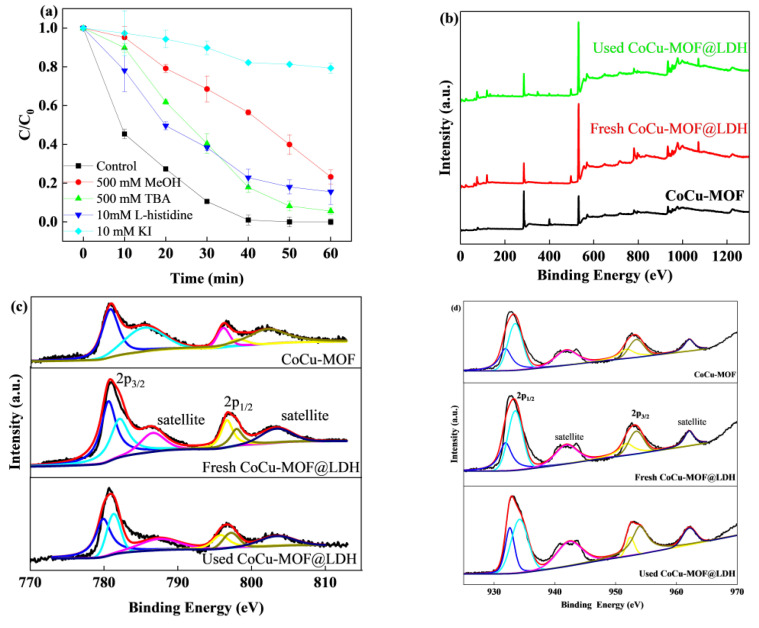

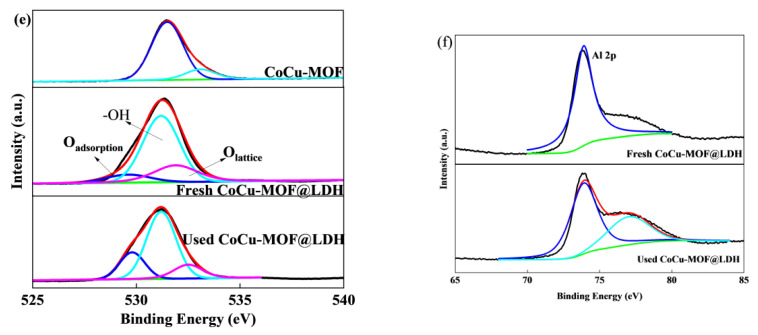
(**a**) The effect of radical scavengers on SMX degradation. (**b**) XPS survey of CoCu-MOFs and CoCu/LDH (fresh and used). (**c**) Co 2p. (**d**) Cu 2p. (**e**) O 1s. (**f**) Al 2p.

To identify the dominant reactive species during the CoCu/LDH/PMS process, radical quenching experiments were performed. As described in previous studies, MeOH was chosen as a scavenger for sulfate and hydroxyl radicals. TBA and L-histidine were used to assess hydroxyl radicals and ^1^O_2_, respectively [48]. The SMX removal slightly decreased in the presence of TBA (94.4%), while the SMX removal declined to 76.8% and 82% in the MeOH and L-histidine quenching experiments, respectively, which was shown in Figure 6a. The results demonstrated that sulfate radicals, hydroxyl radicals and singlet oxygen participated in the CoCu/LDH/PMS process. The relative contributions of the ROS were sulfate radicals > singlet oxygen > hydroxyl radicals. In addition, in the presence of KI, degradation was completely inhibited, indicating that catalysis was more likely to occur on the catalyst surface than in solution [49].

To determine the role of the active sites on the surface of the catalyst, the XPS spectra of CoCu-MOFs and CoCu/LDH (fresh and used) were collected, as shown in Figure 6b–f. The concentration of cobalt with a low-valence state on the catalyst surface decreased from 26.79% to 15.4% after the reaction, while the percentage of cobalt with a high-valence state increased from 28.01% to 32.1%. The results showed that cobalt species underwent a recycling loop on the catalyst surface, where low-valence-state cobalt was renewed during the reaction. Moreover, the relative contents of copper with low- and high-valence states in the fresh CoCu/LDH catalyst were 52.22% and 47.78%, respectively. After the catalytic reaction, the relative contents changed to 37.31% and 62.69%, respectively. Both cobalt and copper species were active sites on the catalyst surface and participated in subsequent PMS activation [50]. Initially, copper and cobalt species with a low-valence state can directly participate in PMS activation through electron transfer to generate sulfate radicals, followed by hydroxyl radicals. On the other hand, copper and cobalt species with high-valence states could also react with PMS to form SO_5_^−^, which could be converted to singlet oxygen via a sequential reaction. Moreover, dissolved oxygen in solution undergo electron transfer with low-valence-state metals to yield O_2_^·−^, which were reduced in the reaction cycle and then further participated in PMS activation [51,52,53]. These metal species also combined with H_2_O/OH^−^, leading to the formation of metal-OH groups on the catalyst surface. These metal-OH groups could react with PMS through hydrogen bonding to produce sulfate radicals through electron transfer. The concentration of O can be evaluated in terms of lattice oxygen and absorbed oxygen. The relative ratios of lattice oxygen to absorbed oxygen were 0.42 and 0.76 before and after the reaction, respectively. Oxygen vacancies were also involved in PMS activation via conversion to active oxygen, resulting in the generation of singlet oxygen.

**Figure 7:**
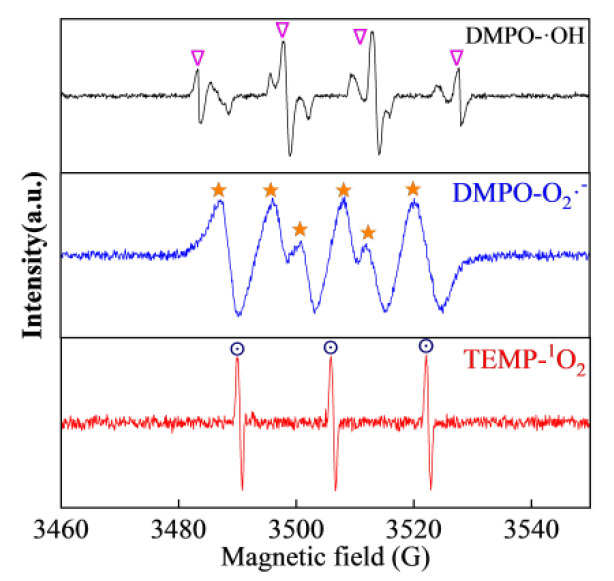
EPR spectra with the addition of DMPO and TEMP. Experimental conditions: [SMX] = 15 mg/L, [PMS] = 0.4 mM, and [catalyst] = 0.1 g/L.

Moreover, the reactive species involved in SMX degradation were investigated by EPR with DMPO and TEMP as spin trapping agents. As shown in Figure 7, no signals were observed with PMS alone in the system. Once CoCu/LDH was added to the PMS-containing solution, strong characteristic signals were obtained, which could be attributed to the presence of DMPO-X, where X represents hydroxyl and sulfate radicals [54]. The signal of DMPO-SO_4_ was confined to the signal of DMPO-X because of its short lifetime in solution and quick conversion to hydroxyl radicals. In addition, TEMP was used to monitor ^1^O_2_. Typical characteristic peaks of TEMP-^1^O_2_ were observed with an intensity ratio close to 1:1:1. High peak intensities were also observed in the reaction, which confirmed the participation of non-radicals, i.e., ^1^O_2_, in the CoCu/LDH/PMS reaction.

**Figure 8:**
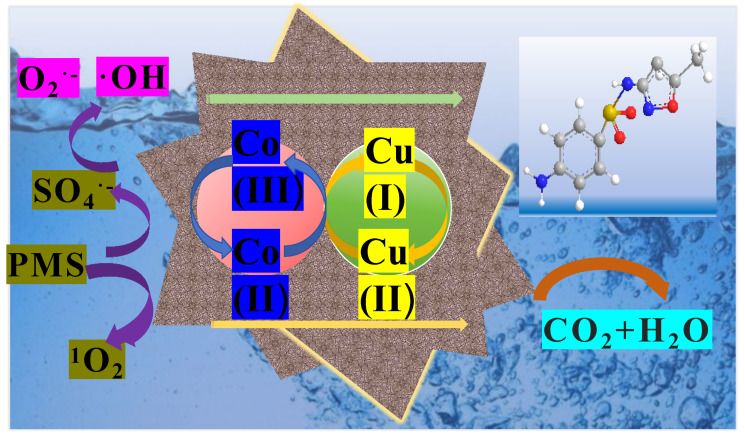
The possible SMX degradation mechanisms in the CoCu/LDH/PMS systems.

On the basis of the above results, sulfate radicals, hydroxyl radicals, and singlet oxygen were produced in the reaction, which involved both radical and non-radical degradation processes [55]. The possible SMX degradation mechanisms in the CoCu/LDH/PMS systems was shown in Figure 8. Initially, sulfate radicals were generated due to PMS activation by the CoCu/LDH catalyst. Moreover, hydroxyl radicals were produced in the solution owing to the conversion of sulfate radicals. Subsequently, electron transfer between Cu(II) and Co(III) occurred, resulting in the formation of Cu(I) and Co(II). Additionally, a non-radical pathway involving ^1^O_2_ also occurred, which might be caused by the dissolved oxygen in solution, lattice oxygen vacancy in catalysts, partial conversion of O_2_^•−^, and the self-decomposition of PMS. At the same time, high-valence-state copper and cobalt ions were reduced in the solution. Ultimately, all the free radicals and non-radical pathways facilitated SMX degradation, converting SMX to byproducts or even completely mineralized to CO_2_ and H_2_O.

Catalyst-Co(II) + HSO_5_^−^ → Catalyst-Co(III)+ SO_4_^•−^ + ^•^OH (9)

Catalyst-Cu(I) + HSO_5_^−^ → Catalyst-Cu(II)+ SO_4_^•−^ + ^•^OH (10)

Co(III) + Cu(I) → Co(II) + Cu(II)(11)

Catalyst-Co(III) + HSO_5_^−^ → Catalyst-Co(II) + SO_5_^•−^ + OH^−^ (12)

Catalyst-Cu(II) + HSO_5_^−^ → Catalyst-Cu(I) + SO_5_^•−^ + OH^−^ (13)

HSO_5_^−^ + ^•^OH→ SO_5_^•−^ + H_2_O (14)

SO_5_^•−^ + SO_5_^•−^→ SO_4_^•−^ + SO_4_^•−^ + O_2_ (15)

O_v_→ O^*^ (16)

HSO_5_^−^ + O^*^→ HSO_4_^−^ + ^1^O_2_ (17)

SO_5_^2−^ + HSO_5_^−^ →HSO_4_^−^ + SO_4_^2−^+ ^1^O_2_ (18)

HSO_5_^−^ → SO_5_^•−^+ H^+^ + e^−^ (19)

2SO_5_^•−^+ H_2_O→2HSO_4_^−^ +1.5 ^1^O_2_ (20)

O_2_ + e^−^ → O_2_^•−^ (21)

O_2_^•−^+ H_2_O → 0.5 H_2_O_2_ + 0.5 ^1^O_2_ + OH^−^ (22)

SO_4_^•−^ + H_2_O →^•^OH + SO_4_^2−^ + H^+^ (23)

(SO_4_^•−^ +/^•^OH+/^1^O_2_ +/O_2_^•−^) + SMX → intermediates + CO_2_ + H_2_O (24)

### 3.6. Possible Degradation Pathways of SMX

**Figure 9:**
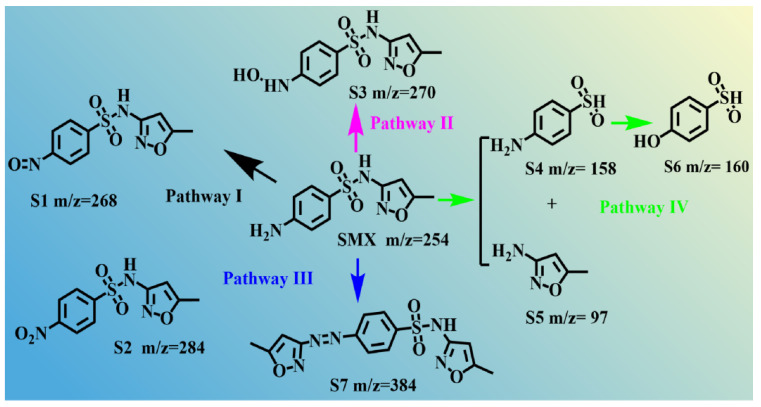
Proposed degradation pathways of SMX in CoCu/LDH/PMS system.

The SMX degradation pathways and intermediates in the CoCu/LDH/PMS activation system were explored via LC/MS, as shown in Figure 9. Table S2 displays the details of the intermediates during the reaction. Four degradation pathways were proposed. In pathway I, the benzene ring undergoes oxidation of the amino group and S1 is formed; then, S2 is produced as oxidation occurs [9]. Similarly, in pathway II, hydroxyl radicals first attack the N atom on the benzene ring of SMX at electrophilic sites, where SMX-OH intermediates are found (S3, *m*/*z* = 270). Then, the N-H bond can be further oxidized to generate S1 and S2. In pathways III and pathway IV, SMX degrades to S4 and S5 through the cleavage of S-N bond, whereas S6 is generated through hydroxyl substitution. S5 can easily react with SMX, leading to the formation of S7. Subsequently, ring-opening, oxidation, and C-N bond cleavage are considered to produce low-molecular-weight chemicals [56]. Finally, the intermediates undergo further oxidation, resulting in the generation of CO_2_ and H_2_O. The positive and negative LC/MS spectra reveal that S1 and S3 are the main intermediates in the CoCu/LDH/PMS activation system. It was considered that pathway I and pathway II are the main degradation pathways in the CoCu/LDH/PMS activation system.

Based on the above results, CoCu-MOFs coated with CuAl/LDH were successfully prepared through a simple synthesis in this work. The CoCu-MOFs nanoparticles confined to the LDH layer not only offer reactive sites or PMS activation but also facilitate the redox recycling of metal species. Different experimental parameters, such as catalyst dosage, PMS concentration, initial solution, pH and common inorganic ions, were investigated, and the CoCu/LDH catalyst could maintain high catalytic activity over a wide pH range due to its unique structure. Radical quenching experiments and EPR analysis indicated the involvement of radical and non-radical degradation pathways. The catalyst exhibited excellent degradation ability, stability, and reusability. The SMX removal remained high after four cycles. Four degradation pathways corresponding to potential intermediates in SMX degradation were explored via LC/MS analysis.

## 4. Conclusions

The CoCu/LDH catalyst exhibited good catalytic activity and universality for other target pollutants with limited metal leaching. However, impurity peaks were still observed in the XRD patterns. The yield of the catalyst is still unsatisfactory, which will lead to high costs in practical applications. Notably, the SMX removal in solution reached 100%. However, the TOC removal in this system was 53.7%, which demonstrated that low-molecular-weight intermediates remained in the solution. The radical quenching experiment proved that the CoCu/LDH catalyst produced both sulfate radicals and hydroxyl radicals during the PMS activation. Moreover, the CoCu/LDH catalyst showed excellent stability over four runs. With the nanoparticles confined in densely stacked lamellas, the catalyst promoted the PMS activation for SMX removal. This work illustrated a simple and effective method of preparing a core-shell CoCu/LDH catalyst and opens a new direction for its actual application in environmental remediation.

## Data Availability

The original contributions presented in this study are included in the article/Supplementary Materials. Further inquiries can be directed to the corresponding author.

## Supplementary Materials

The following supporting information can be downloaded at: https://www.mdpi.com/article/10.3390/nano15060432/s1, Table S1: Studies on various contaminants using MOF-based and LDH-based catalysts in a heterogeneous Fenton reaction; Table S2: SMX degradation intermediates by LC/MS. References [57,58,59,60,61] are cited in the supplementary materials.
