# Supplementary material for: Densely Stacked CoCu-MOFs Coated with CuAl/LDH Enhance Sulfamethoxazole Degradation in PMS-Activated Systems
Source: Nanomaterials (Basel). 2025 Mar 11;15(6):432. doi: 10.3390/nano15060432 (PMC11946684; doi:10.3390/nano15060432)
Supplement: Supplementary file 1 [file nanomaterials-15-00432-s001.zip › nanomaterials-3481136-supplementary.pdf]

Densely Stacked CoCu–MOFs Coated with CuAl/LDH Enhance Sulfamethoxazole Degradation in PMS-Activated Systems

Xin Zhong [1,*], Xiaojun Liu [2], Meihuan Ji [1] and Fubin Jiang [2]

[1] Experimental Education Platform, Beijing Normal University at Zhuhai, Zhuhai *519087*, China; meihuanji@bnu.edu.cn
[2] Faculty of Arts and Sciences, Beijing Normal University at Zhuhai, Zhuhai *519087*, China; xiaojunliu@bnu.edu.cn (X.L.); jfb@bnu.edu.cn (F.J.)
* Correspondence: zhongxin@bnu.edu.cn; Tel.: +86-0756-3621560

Table S1. Studies on various contaminants using MOF-based and LDH-based catalysts in a heterogeneous Fenton reaction.

No.	Catalyst	Target pollutant	Time efficiency	Operation parameters
1	MgAl/LDH-coated MOF-derived $Co_{2.25}Mn_{0.75}O_4$	SMX	60 min; 100%; 68.3% (TOC)	PMS concentration: 0.05 g/L; catalyst dosage: 0.1 g/L; SMX concentration: 3 mg/L; reaction temperature: 25 ◦C
2	MOF/LDH-derived heterostructured $Co_3O_4/MnCo_2O_4$ composite	Levofloxacin	30 min; 96.9%	Catalyst: 40 mg/L; LEV concentration: 10 mg/L; PMS concentration: 0.3 g/L; pH: 6.5; reaction temperature: 25 ◦C
3	Ni-MOF-derived NiCo-LDH	RR-120 dye	10 min; 89%	PMS concentration: 3 mM; catalyst dosage: 5 mg/L; dye concentration:$1*10^{-4}$ M
4	MOF-derived CuCo carbon microspheres assembled with nitrogen-doped carbon nanotubes	P-nitrophenol	30 min; 100%	T = 25 ◦C; [PNP] = 5 mg/L; [PMS] = 1.2 mM; [catalyst] = 30 mg/L)
5	MOF-derived carbon-supporting CuCo nanospheres	Orange G	15 min; 100%	OG = 10 mg/L; catalyst dose = 50 mg/L; PMS concentration = 2 mM
6	MOF-derived porous Fe-Cu@carbon	Bisphenol A	10 min; 100%	Catalyst dosage: 1.0 g/L; BPA concentration: 50 mg/L; PS concentration: 0.028 mM; pH 5.0
7	Fe-Cu–layered double	Butyl xanthate	10 min; >90%	Catalyst dosage: 0.2 g/L; BX concentration: 20 mg/L;

	hydroxides/biochar composites			PS concentration: 0.1 mM
8	$Cu_2Fe_{0.5}Al_{0.5}$-LDH	SMX	10 min; 98.25%	Catalyst dosage: 0.1 g/L; SMX concentration: 10 mg/L; PMS concentration: 0.3 mM
9 This work	Densely stacked CoCu-MOF coated with CuAl/LDH	SMX	60 min 100%	Catalyst dosage: 0.1 g/L; PMS concentration: 0.4 mM; SMX: 15 mg/L

[1] Su, C.; Zhang, N.; Zhu, X.; Sun, Z.; Hu, X. pH adjustable MgAl@LDH-coated MOFs-derived $Co_{2.25}Mn_{0.75}O_4$ for SMX degradation in PMS activated system. Chemosphere. 2023, 339, 139672.

[2] Deng, Q.; Zhang, X.; Chang, L.; Chai, H.; Huang, Y. The MOF/LDH derived heterostructured $Co_3O_4/MnCo_2O_4$ composite for enhanced degradation of levofloxacin by peroxymonosulfate activation. Separation and Purification Technology, 2022, 294, 121182.

[3] Ramachandran, R.; Sakthivel, T.; Li, M.; Shan, H.; Xu, Z.; Wang, F. Efficient degradation of organic dye using Ni-MOF derived NiCo-LDH as peroxymonosulfate activator. Chemosphere. 2021, 271, 128509.

[4] Shi, Q.; Hou, Y.; Zhu, Q.; Hao, Y. MOF-derived CuCo carbon microspheres assembled with nitrogen-doped carbon nanotubes as PMS activator for the efficient degradation of p-nitrophenol. Separation and Puriɜcation Technology, 2025, 354, 129107.

[5] Li, H.; Su, L.; Zheng, J.; Lu, S.; Yang, Z.; Wang, C.; Xu, S.; Zhou, Q.; Tang, J.; Huang, M.; Zhang, Y. MOFs derived carbon supporting CuCo nanospheres as efficient catalysts of peroxymonosulfate for rapid removal of organic pollutant. Chemical Engineering Journal, 2023, 451, 139114.

[6] Garcı́a, A.; Sanchez, N.C.; Palomino, G.T.; Cabello, C.P. MOF derived porous Fe-Cu@carbon catalyst for the degradation of bisphenol A through a persulfate-based advanced oxidation process. Microporous and Mesoporous Materials, 2025, 381,113366.

[7] Li, B.; Hu, X.; Gu, Y.; Zhang, W.; Xu, W.; Yang, H.; Ye, S.; Yang, Z.; Liu, N.; Tan, X. Complexation between butyl xanthate and Cu enhanced peroxydisulfate activation and cation redox cycle by Fe-Cu-LDH/biochar. Journal of Environmental Chemical Engineering, 2024, 12, 112466.

[8] Xin, Q. Liu, S.; Lu, S.; Chen, Z.; Han, P.; Xin, S.; Wang, Q.; Liu, G.; Zhou, C.; Xin, Y.; Yan, Q. Surface-bound sulfate radical-dominated degradation of sulfamethoxazole in the CuFeAl-LDH/peroxymonosulfate system: The abundant hydroxyl groups enhancing efficiency mechanism. Chemical Engineering Journal, 2023, 471, 144453.

Table S2 SMX degradation intermediates by LC/MS

Name	m/z	Chemical formula	LCMS spectra
SMX	254	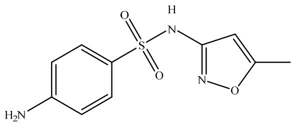	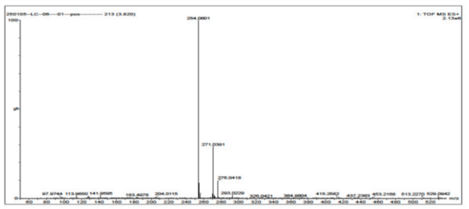
S1	268	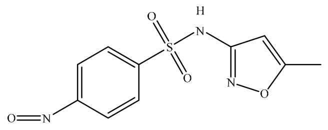	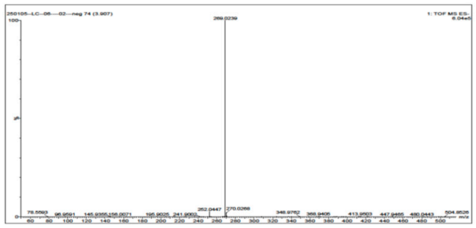
S2	284	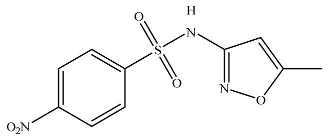	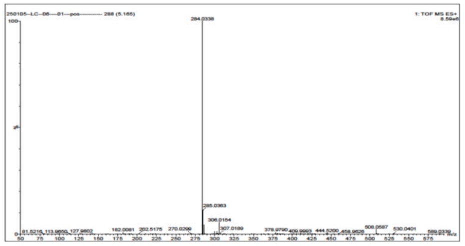
S3	270	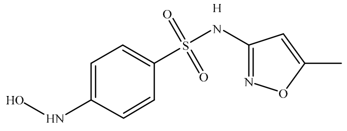	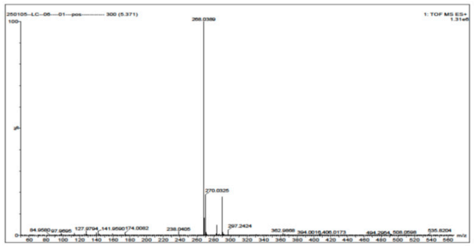
S4	158	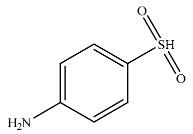	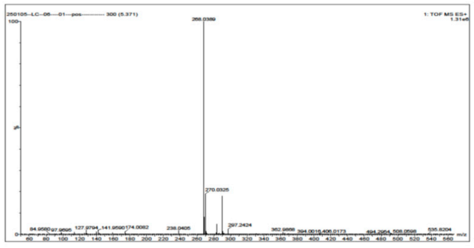

S5	97	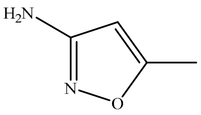	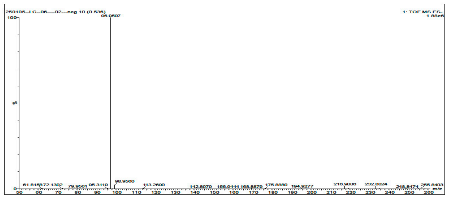
S6	160	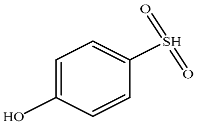	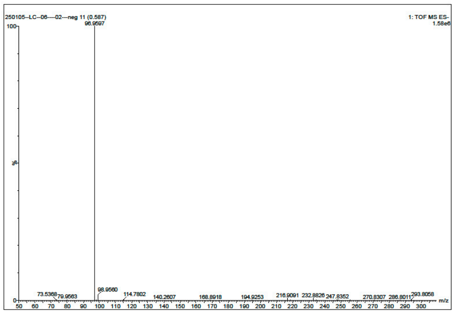
S7	384	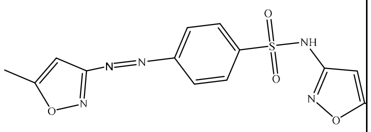	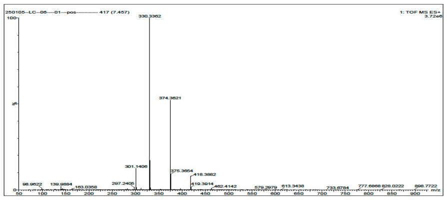
